# Identity text: an educational intervention to foster cultural interaction

**DOI:** 10.3402/meo.v21.33135

**Published:** 2016-11-01

**Authors:** Zareen Zaidi, Daniëlle Verstegen, Rahat Naqvi, Tim Dornan, Page Morahan

**Affiliations:** 1 Division of General Internal Medicine, Department of Medicine, University of Florida, Gainesville, FL, USA; 2 Department of Educational Development and Research, Faculty of Health, Medicine and Life Sciences, Maastricht University, Maastricht, The Netherlands; 3 Languages and Diversity, University of Calgary, Calgary, AB, Canada; 4 FAIMER Institute, Philadelphia, PA, USA; 5 Department of Microbiology and Immunology, Drexel University College of Medicine, Philadelphia, PA, USA

**Keywords:** educational cultural hegemony, pedagogical space, cross-cultural education, sociocultural theory, discourse analysis

## Abstract

**Background:**

Sociocultural theories state that learning results from people participating in contexts where social interaction is facilitated. There is a need to create such facilitated pedagogical spaces where participants can share their ways of knowing and doing. The aim of this exploratory study was to introduce pedagogical space for sociocultural interaction using ‘Identity Text’.

**Methods:**

Identity Texts are sociocultural artifacts produced by participants, which can be written, spoken, visual, musical, or multimodal. In 2013, participants of an international medical education fellowship program were asked to create their own Identity Texts to promote discussion about participants’ cultural backgrounds. Thematic analysis was used to make the analysis relevant to studying the pedagogical utility of the intervention.

**Result:**

The Identity Text intervention created two spaces: a ‘reflective space’, which helped participants reflect on sensitive topics such as institutional environments, roles in interdisciplinary teams, and gender discrimination, and a ‘narrative space’, which allowed participants to tell powerful stories that provided cultural insights and challenged cultural hegemony; they described the conscious and subconscious transformation in identity that evolved secondary to struggles with local power dynamics and social demands involving the impact of family, peers, and country of origin.

**Conclusion:**

While the impact of providing pedagogical space using Identity Text on cognitive engagement and enhanced learning requires further research, the findings of this study suggest that it is a useful pedagogical strategy to support cross-cultural education.

In health professions education, new academic partnerships between North American and European institutions and health centers in developing countries have emerged (1, 2). This globalization is ushering in an increasingly interconnected world with complexities for cross-cultural communication in both face-to-face and online settings (3, 4). Exploring and understanding cultural difference have been noted as critical for establishing trust and determining long-term success of educational programs (1, 2, 5, 6). It is of note that when trust is undermined in multicultural learning environments, learners from minority backgrounds have reported increased depression, anxiety, hypertension, cardiovascular, pulmonary, and pain conditions (7–9).

Dogra and colleagues have recently published AMEE Guide No. 103 for assisting faculty in designing, delivering, and assessing diversity curricula (10). They note that ‘course design is not value free or unbiased as it is dependent on the perceptions held by educators’ and that many educational approaches are ‘rooted in the historical context of white domination of disadvantaged minorities and are very race or ethnicity focused’. This conclusion emphasizes research, which has shown that groups in power may dictate assumptions about culture, leading to ‘common-sense understandings that serve for all’ (11). Such ‘dominant discourse’, the way of speaking created by those in power, thus becomes the accepted way of looking at or speaking about the subject (12). The phenomenon of stifling of non-dominant discourses by a tacitly dominant discourse is termed ‘cultural hegemony’ (13, 14). ‘Educational cultural hegemony’ occurs when teachers assume that content and task are culture free, and unconsciously, implicitly discourage introducing the student’s personal cultural context. Markus and Conner have highlighted numerous examples where educational methods have been dominated by teachers from Western independent cultures, inhibiting the engagement and learning by those from interdependent cultures (6).

One of Dogra and colleagues’ key recommendations is: ‘provide a safe learning environment but be prepared to challenge students to push themselves’ (10). Our earlier research emphasizes this challenge. We found that health professions educators from diverse cultures who were participating in residential program sessions, followed by online distance educational discussions, made surprisingly few references to sociopolitical events or norms in their home countries (15). When they did, their online contributions were more likely to be greeted by silence or superficial, short-lived discussions than in-depth exploration of the issues raised.

Educational literature reveals the need for ‘pedagogical space’ to promote a safe place for sense making of such exchanges about culture and identity that can foster learning (16, 17). Pedagogical spaces are not only physical, but also narrative social spaces with historical and cultural dimensions in which learners interact (18). Sociocultural theorists and practitioners have described learning in collaborative cultural contexts (19) as well as a lack of facilitating interactional, narrative pedagogical space (20). The gap we identify in the literature is the lack of specific educational interventions to provide safe pedagogical space that can promote cross-cultural exchanges.

Creation of ‘Identity-Safe Classrooms’ where teachers encourage discussions about learners’ identities has been shown to improve student performance on standardized testing (21). Cummins and Early have described Identity Text as an educational strategy that promotes this pedagogical space (22). Identity Text is an intervention, orchestrated by the teacher or facilitator, to describe and discuss learners’ creative work, which can showcase the influence of cultural background on the individual (23). Identity Text engages learners by asking them to create ‘texts’ (e.g., creative writing and other multimodal forms of cultural production) that express the identity or influence of cultural background on the individual in a new social setting (22). Identity Text thus offers a method to challenge hegemonic societal trends by bringing learners’ cultural backgrounds to the foreground, and drawing attention to the multiple facets of life experiences which shape interactions in learning environments (6, 23). While Identity Text has been described in K-12 educational settings (22), in this study we explored the use of the Identity Text intervention in a very different setting. The objective of this research was to evaluate the use of Identity Text as a structured educational intervention to promote discovery and dialogue about participants’ cultural backgrounds. Specifically, this project set out to describe the application of the intervention in an educational context that comprises an asynchronous online platform and global health professions education faculty development program.

## Methods

### Theoretical framework

Our aim is to introduce Identity Text as a pedagogical tool for sociocultural interaction, which places this research within the critical theory paradigm (24). Discourse theory (25), which falls in the scope of critical theory, posits that language is not only about what the person is *saying* (informing others) but also what the person is *doing* (actions) and *being* (identity). Language can exercise and resist power (25). It is ‘never neutral’ (12) because it incorporates tacit assumptions of what is normal and right (25). Qualitative data analysis, from this standpoint, can identify power relations and cultural assumptions (12, 26).

### Context

The US-based Foundation for Advancement of International Medical Education and Research (FAIMER^®^) has a 2-year global fellowship program for midcareer health professions faculty from over 40 developing countries (5). The program goals are to strengthen knowledge and skills in education, leadership, and project management, and build a community of practice in health professions education with the aim of improving the health of communities (5). The program comprises an initial 3-week face-to-face immersion residential session in Philadelphia, an 11-month e-learning period with online, asynchronous discussions (conducted via listserv), a second 2-week residential session, and a second and final 11-month e-learning period. Participants remain members of the community of practice after graduation and continue to take an active role in online discussions.

### Intervention

During 2013–2014, the FAIMER Institute invited alumni from the years 2001 to 2011 to convene a 2-week ‘FAIMER Community Conversation’ on a topic of their choice. ZZ (FAIMER Institute Fellow alumnus) convened a conversation in December 2013 entitled ‘Identity Matters’ and invited the coauthors as guest faculty. We used Identity Text (27) as the educational intervention because it fits with a learner-centered approach and stimulates constructive, contextual, and collaborative learning. We asked participants to actively connect the topic of identity to their own daily life and history (constructive and contextual) and reflect on the Identity Text of others (constructive and collaborative) (28).

### Data gathering process

ZZ asked FAIMER listserv participants to submit an Identity Text describing how their identity as an educator had evolved over time. The written cue (Appendix 1) asked them to take into account how the people, traditions, politics, language, religion, race, ethnicity, gender issues, and economy of their country had influenced their identity as a person and educator. It asked participants to be creative in their submission, using any medium of communication (e.g., art, poetry, slides, and videos). ZZ then facilitated the online discussion about how participants’ cultural backgrounds influenced their roles as educators and leaders. As part of the collaborative approach, the authors also contributed their own Identity Texts and joined in the discussion. At the end of the 2 weeks, ZZ compiled the submitted Identity Texts into a 109-page text, which served as the data set for analysis.

### Critical reflexivity

The authors have diverse backgrounds: ZZ is a FAIMER Institute Fellow alumnus, who trained as an internist in New York and emigrated from Pakistan to the USA. DV is Dutch, lived in Asia and Italy, and works with learners from all around the world at Maastricht University. RN lived in France while obtaining her PhD, immigrated to Canada from Pakistan, and focuses on language and diversity education. PM is an American, founding co-director of the FAIMER institute, and has worked to promote gender and minority issues. TD works in the UK, has lived in the Netherlands, is an expert in critical research, and has worked with health professions educators from cross-cultural backgrounds. These authors took part in a conscious explicit process of critical reflexivity, discussing via Skype calls and emails how their backgrounds might influence analysis of the texts. They discussed their preconceptions and interpretations of the data, using their diverse backgrounds as resources to provide cultural insights. ZZ, DV, PM, and TD participated actively by submitting their own Identity Texts online in order to make transparent their own subjectivities, to foster a safe pedagogical space and enhance critical reflectivity.

### Data analysis

This was a thematic analysis, which used Braun and Clarke’s framework of latent thematic analysis to analyze the Identity Texts (29). Bearing in mind the research question and following the six phases described by Braun and Clarke, two of the authors (ZZ and RN) independently analyzed the data and identified themes, focusing on patterns and richness of responses rather than the number of responses, and assigned comments to themes. ZZ then wrote a narrative of the results, proceeding from identification of cultural themes to analysis of the content of the themes to synthesis and explanation. A latent thematic analysis goes beyond semantic content of data and identifies underlying ideas and assumptions (29). To study these broader meanings, we used a set of analytical tools described by Gee, which are compatible with our critical theoretical orientation, to identify typical stories or figured worlds; these are narratives and images that different social and cultural groups use to make sense of the world (25). They function as simplified models of how things work when they are ‘normal’ and ‘natural’ from the perspective of a particular social and cultural group that the participants invited listeners to assume (30). Gee’s tools were also used to study how participants’ language enacted distinctive ways of interacting, valuing, feeling, and believing (31). All authors contributed to the evolving narrative, and an audit trail was maintained.

## Results

Twenty-eight participants, including four of the authors, contributed to the online discussions. Participants who posted their Identity Texts came from 11 countries in Africa (Ethiopia, Egypt, and South Africa), Asia (India, Pakistan, Turkey, Bangladesh, China, and Philippines), and Latin America (Brazil and Colombia). Three co-authors from the US, Netherlands, and UK, and the first author from Pakistan also contributed their Identity Texts.

Our analysis revealed that the Identity Text intervention provided submissions about the impact of sociocultural factors on the formation of identity that could be framed as two pedagogical spaces: a ‘reflection space’ and a ‘narrative space’. Appendix 2 contains verbatim examples to illustrate what we mean by those terms. Below, we present an analysis of the text in these two spaces.

### A reflection space

Participants used the Identity Text framework to reflect on tensions they faced in their life and careers. Several frank and open discussions emerged on sensitive topics such as institutional environments, roles in interdisciplinary teams, and gender discrimination. Several participants commented that they were ‘happy to have this opportunity’ which invited ‘introspection’, ‘encouraged them to share their story’ and discuss topics that were often in their subconsciousness or for which they had no space to discuss in an educational setting.

#### Identity dissonance

One topic of reflection was on tensions between traditional academic and actual pedagogical responsibilities. One participant described contrasting figured worlds where ‘an identity has been built … that promotion is always and only possible through publication or research’. Life as a university professor was compared metaphorically to ‘soccer only targeted at scoring goal but not showing a beautiful game or the art of soccer (targeted at publishing not the art of teaching)’ (Participant A, Ethiopia).

A second topic of reflection was the tension caused by power relations and the struggle with dominant perceptions about who holds authority in interdisciplinary teams, which was highlighted by two participants who were nurses. They described dissonance related to explicit expectations and actual experience, and to theoretical status and practical hierarchical work environments. One nurse described how she was introduced as ‘Doctor’ as ‘a quick way to gain respect from the people’: ‘when I said or did something impressive then I wanted people to know that even a nurse can think and advocate for their health; and I did that thinking this was my contribution to nursing’ (Participant B, Pakistan). The text refers to unspoken assumptions about the role of a nurse and the role of a doctor, that is, a doctor can go into a nurse’s space but not the reverse.

#### Gender-related tensions

Participants also reflected on tensions faced around gender-related societal expectations, which in some parts of the world still determine career choices. A participant from India highlighted the expected roles of women as child bearers rather than doctors, although this expectation might not be openly acknowledged by society (Participant C, India). Her response brought to light the tensions faced by women in India as wives, mothers, and women physicians. Participants from developed countries also described gender-related stereotypes. As an example, one participant shared that her mother had said: ‘When you have a family, you will soon stop working again to care for your children’ but ‘luckily, my current world allows me to combine both’ (Participant D, Netherlands). She wrote that she had ‘also refused to accept that women should have different role in society’. This participant also noted that she was one of eight women of the 130 researchers in her institute, and ‘how some of those eight fought to be more male than male, others disappeared into the background, and a third group overexposed their femininity. None of them attractive choices’ (Participant D, Netherlands). Another participant drew the group into an academic figured world in the 1980s when she ‘was one of less than 150 women chairs in either basic or clinical science across the US’. She described pushing this into her subconsciousness for many years and having gender bias pointed out to her during a leadership fellowship program, when ‘some faculty looked to the men Fellows rather than the women Fellows when answering questions’. Understanding the ‘gender bias’ over the years and ‘reflection on this experience led me to assume my identity in advancing women!’ (Participant E, USA).

In these examples, participants reflected on tensions in careers, notably their perceptions about academic versus pedagogical responsibilities, power relations in interdisciplinary teams, and gender biases. Through these reflections facilitated by the Identity Text strategy, participants were not just ‘saying’; they were also ‘doing’. Through their reflections, they were educating others about struggles they faced in academic advancement in their particular cultural settings.

#### Role of middle-class culture

Participants described how middle-class culture in their countries led them to choosing medicine as a career: ‘Society, peers, and family endorsed joining a prestigious and well-paying course of medicine’ (Participant C, India). Another participant stated that being told from an early age that she ‘must’ become a doctor led her to become a doctor. She too attributed this to her ‘middle-class background’ (Participant F, India).

### A narrative space

#### Role of social network on identity formation

Our analysis of the Identity Texts also revealed powerful stories about how participants saw and understood the world, a narrative space. Several participants wrote retrospectively in a storytelling fashion, building connections, on the role family members played in shaping their careers. One participant used the metaphor of dance to describe the role of parents as mentors: ‘In my dance of life, from my father I got the steps of inquiry, a love for reasoning, and the heated side of my temperament that will stand me in good stead for the tango or the Pasodoble. My mother’s contribution is linked rather to the more subtle dances like the rumba and the waltz’ (Participant G, South Africa). The participant appeared to be making explicit information that might otherwise be buried in the subconsciousness.

A participant contributed her story which traversed three generations of musicians at the University of the Philippines Conservatory of Music and that coming from a well-known musician family ‘genetics has something to do with the love of teaching’ (Participant H, Philippines). She herself went onto a career in medicine but noted the influence her mother had on her career:

listening to her colleagues, students and friends recount their most memorable experiences with my mother, I am once again awed by her courage, her commitment and dedication to parenting and teaching, her simplicity, her integrity and her passion for excellence in everything she did. Thank you, Mom. I hope that we inherited those good traits.

Participants discussed fortuitously meeting mentors who shaped their careers. Incorporation of ‘love for dissection’ and anatomical pathology, passion for microbes, interdisciplinary research, health professions education, and leadership stemmed from mentorship, as one participant described: ‘We instinctively identify with particular individuals and tend to model our behavior and activities on them, either unconsciously, or consciously and deliberately, aided by a process of reflection’ (Participant G, South Africa).

#### Role of politics on identity formation

Participants used Identity Text to share stories that were quite specific to their particular cultural environment. As an example, the above participant also articulated a figured world of South Africa emerging from apartheid, explained the health professions context, and provided an ‘insider view’ of the impact of political events. Her story demonstrated historical cultural experiences related to race, socioeconomic factors that have played a critical role in shaping her worldviews, and provided a rich resource to discuss coexistence with various cultural backgrounds:

the uprising of school children against the form of education to which they were subjected was a watershed moment in the transition to the democracy finally achieved in 1994. So the political landscape had a major influence to my growth and development. (Participant I, South Africa)

#### White western view

A western faculty member described her struggle in a cross-cultural teaching environment as, on one hand, ‘being quite comfortable in an outsider position’ and, on the other hand, not being able to connect with women from other cultures outside the work environment:

I was really eager to talk to the Moroccan and Turkish mothers of my son’s class mates, but we had little to discuss. They did not work and I had no religion. We talked about our children, but that was about it. I was quite disappointed with myself for not being able to connect. (Participant D, The Netherlands)

She went on to draw us into the figured world of a western faculty member in a cross-cultural learning environment looking ‘for borders between two fields’ where ‘volunteering my white-western view instead of waiting for your discussion points’ felt ‘like an elephant in the china cabinet’ (Participant D, The Netherlands).

Another white participant from South Africa commented on how seemingly ‘fragile’ partnerships at academic institutions administratively controlled by minority white faculty continue to work and how she found ‘the dynamics in the school, which had lecturers of all races, were fascinating’. She also realized how isolated she had been from the social and political realities of South Africa: ‘how my own thinking had also been stifled by the system and how liberating it was to have access to a much wider cultural spectrum’ (Participant I, South Africa).

A white faculty member from the USA commented on how using ‘Identity Text’ reminded her of race-related conversations she had taken part in and how such conversations help unpack ‘unearned privileges’ like being white and male (Participant E, USA).

## Discussion

### Principal findings

Our research demonstrates that the Identity Text (23) educational intervention can provide formal, legitimate ‘pedagogical space’ that facilitates cross-cultural education in an online global faculty setting where many participants have not personally met each other. Our analysis of the submissions revealed two complementary components of that legitimized space. The reflection space enabled learners to reflect on deep cross-cultural issues such as power dynamics, gender bias, and tensions between being an academic and educator. The narrative space fostered the exchange of thoughtful stories about personal backgrounds and local situations. The reflection and narrative space provided space to discuss issues such as gender, politics, and white privilege, which are generally not brought up in learning environments. Cultural hegemony in health professions education inadvertently encourages learners to leave their cultural backgrounds at the classroom doorstep (32). Identity Text helps challenge these hegemonic trends by providing a safe space to share and learn about these important issues. These discussions are critical in medical education because of two main reasons: first, learners who are adept in multicultural conversations report improved preparedness to take care of diverse population which is associated with improved access to health care for racial and ethnic minority patients, greater patient choice and satisfaction, and better educational experience for health professions students (33, 34). Second, there is now a vast body of literature that challenges ‘color-blind’ policies at institutions (35) as by ignoring racial identities individuals and institutions place minority groups at a disadvantage at the recruitment, retention, and promotion levels (36, 37). Creating forums or hosting focus groups to hear the voices of individuals of color, women, and lesbian, gay, bisexual, and transgender (LGBT) prevent them from feeling isolated and devalued. Supporting and nourishing minority meetings and groups on campus or at an institution increases multicultural interaction, provides a support system, and sends a strong message about the organizational climate (36).

### Relationship with other research

The results indicate that the Identity Text teaching method encompasses many of the methods suggested by Dogra and colleagues to foster diversity competence outcomes such as the ability to ‘evaluate your own attitudes and perceptions (including personal biases) of different groups within society’ and ‘reflect on the relevance of diversity in health and delivery of services’ (10).

Educational researchers have noted a lack of facilitating interactional, narrative interventions (20). Identity Text appears be a simple educational intervention that provides and legitimatizes the pedagogical space that has been noted to be important in promoting sense making (16, 17). It also provides ‘narrative space’ for self and telling one’s stories (38) and ‘reflection space’ to foster reflection in an interactional way.

There are several ways in which this educational intervention may be particularly useful with the increasing number of cross-cultural educational settings and use of distance learning. First, stories provide insights into others’ culture and lead to better understanding and cultural tolerance (16). Moreover, knowing each other’s stories makes participants in a teaching/learning setting feel they are part of a group, which can stimulate engagement, build trust, and reduce dropout rates (39).

Second, it is also important to understand and develop our story about our backgrounds and culture for successful development of professional identity (20). Identity Text, as indicated in some of the participants’ contributions in the results, created an opportunity to discuss tensions and dissonance encountered during professional development. Telling and re-telling personal stories enables learners to understand the development of their professional identity in the sociocultural context (20).

Third, reflection on how individuals position themselves in relation to dominant institutional or social bodies can help learners from minority groups to shed light on their evolving professional identities (40–42). Another educational arena that may be a fertile ground for use of Identity Text is bridging the gap between the professional and personal worlds; this is coming increasingly to the forefront in intergenerational differences in health professions education (43). Finally, the use of Identity Text may facilitate discussions about how cultural context influences education, thus helping to promote more effective implementation of educational theories and research.

### Strengths and limitations

We have demonstrated the feasibility of the Identity Text teaching method in eliciting substantial reflection and stories about educators’ professional development – in the challenging context of an asynchronous online setting, with global learners, many of whom have never met personally.

This study has several limitations. First, participation was voluntary; therefore, those who took part may ‘believe’ in the importance of promoting discussion about the learner’s cultural background. We did not capture the point of view of participants who may not believe or who felt uncomfortable discussing their background in a public forum; for example, this type of disclosure may not be common in some cultures (6). Nevertheless, we had participants from 14 countries in five continents. Second, because this was an exploratory study to establish if Identity Text was a feasible teaching method to elicit submissions and dialogue about cultural issues in a challenging environment (online, global, and lack of personal connections), we did not gather data regarding the ability of Identity Text to result in cognitive engagement and learning, or assess the amount and quality of reflection or narratives. However, Dogra and colleagues have suggested possible assessment methods, which can be incorporated into Identity Text (10).

### Learnings and implications for future practice

We propose the use of Identity Text as an educational intervention that can result in engagement of learners through identity affirmation and building a learning community, which in turn would result in cognitive engagement. We have also identified several strategies for successful use of Identity Text. First, there is the need for practice and experience in preparing thoughtful Identity Texts. We provided one text and two PowerPoint examples for the participants along with the written cue. Second, we found active facilitation was necessary, both to encourage provision of Identity Texts and to encourage reflective reactions to those posted. The German Society for Medical Education has recently proposed specific competencies for faculty to demonstrate their social and communicative ability, which include establishing a working climate conducive for learning and cooperation (44). Although participants submitted Identity Texts, dialogue around these submissions was limited; even with active facilitation, there was variable back-and-forth discussion among participants about the commonalities, differences, or impact of sharing Identity Texts. Finally, educators need to give careful thought to cultural considerations about societal norms about personal disclosure (6). Having faculty provide their own stories, and disclosing their own experiences and biases may be needed to provide a safe space for sharing.

As Dogra and colleagues have indicated (10), further research is needed to measure the long-term impact of teaching methods, such as using Identity Text, on enhanced engagement, learning and building, and sustaining a community of practice (19). Further research is also needed to determine whether an intervention such as Identity Text increases dialogue on sociocultural issues in a professional development setting (15).

## *Appendix 1*. The written cue.

Tell us about the evolution of your identity over time as an educator. Each one of you brings with you a wealth of information about your country; the people, traditions, politics, language, religion, race, ethnicity, gender issues, and economy of the area. Each of these factors has likely influenced you as a person and as an educator. Let’s talk about how these factors affected the evolution of your identity. Attached are two PowerPoint presentations and a write-up for inspiration, which may give you some ideas. Feel free to use any form to communicate your thoughts, including PowerPoint, YouTube, Art, Poetry, Sketches or Pictures (you are not limited to this list). If you can use your native language and translate, that would be great! Try to tell your story, that is, use a narrative style.

## *Appendix 2*. Excerpts from Identity Texts.

- I contemplate the emergence of my personal dance and I see it rooted in the exuberant, provocative, slightly defiant Sophiatown jive, with a good dose of kwaito, a little bit of samba and quite a few steps that I cannot yet predict. I trust in the *emergence* of the normative truth that how we teach and the way we are have an impact on who our students will become and where they will go. My future is a ‘lucky packet’ and still holds many surprises, but my subjective truth is that, as long as my feet keep moving and I am engaged in understanding learning and in guided reflection, my personal insight, my teaching and the meaning of my life will blossom.
- To cut a long story short, I moved from one state to another, mostly in South and Central India, every six years. It meant learning a new language, joining a new school, making new friends, and adapting to a new culture. In each place, while I felt a part of the culture, I was considered an outsider. The funny part is I never stayed in Bihar, so I never identified with it. Today, when someone asks me where I am from, I have a tough time explaining. I feel like saying ‘pan-Indian’. I find it easier to explain to a foreigner that I am Indian, than to my countrymen! And though I feel at home everywhere, I can’t say I am accepted as one of their own anywhere within India. Yet, in all honesty, I cannot say that I have ever been denied any opportunities or rights because of this ‘identity crisis’. I have blended in quite easily everywhere.
- *Dance macabre*. Who does not remember the angst accompanying that first cut through the skin in the dissection hall. ‘Will I recognise the brachial plexus before I destroy it?’ Quickly followed by: ‘Oh my God, I completely destroyed it’, and the embarrassing realisation days later that the brachial plexus is so big that it is impossible to miss. But through all of this we had Dr. ND. She had the grace not to laugh in our faces, but gently guided us to discover all the truths of the body; to develop a love for the gentle dissection of the thalamus; to envision the relationships in the anterior mediastinum, always showing respect towards our cadaver, which so easily could have been flippantly dismissed. I developed such a love for anatomy that I again turned tutor, and AD, my dear friend to this day, still acknowledges my tutoring as the only reason he passed anatomy. Dr ND – a role model and mentor to honour and remember.
- I have been a member of several ‘conversations on race’, ‘healing the wounds of racism’, etc. over the past 30+ years. Much of this work has been ‘identity text’ – to listen and understand where individuals come from, and understand my own identities. For example, in the US there are various ‘unearned privileges’ that tend to come with birth or life stage, being: a man, white, heterosexual, able-bodied, Christian, English speaking, higher socioeconomic status, between 20–60 years old. So on the one side, I am white, so I have unearned privilege; on the other side, I am a woman, so I have less unearned privilege than a man.
- I was born in a middle class family in India and by default, it is the dream of every middle-class parent in India that all their children should become either doctors or engineers. So, they would push and push their children with extra classes, tuitions, medical entrance preparations from Grade 9 onwards etc. In keeping with this tradition, I was told from my childhood that I ‘must’ become a doctor, which I took very seriously and achieved it. So my identity a doctor was partly contributed to by my middle-class culture.
- We were in a strange and uniquely South African situation of being a medical school, which was only for people of colour who came largely from very economically disadvantaged backgrounds. The dynamics in the school, which had lecturers of all races, was fascinating and I realised very early on how isolated I had been from the social and political realities of South Africa, how my own thinking had also been stifled by the system and how liberating it was to have access to a much wider cultural spectrum. The potential for cultural hegemony was obviously very high, the curriculum and accreditation processes and control over who could be admitted to the school and who could teach, were largely held by the White minority authorities, but somehow there was a partnership, sometimes fragile but overwhelmingly honest, between faculty and students.
